# RNAi-based motility screen of predicted sarcopenia-associated genes during aging in *C. elegans.*

**DOI:** 10.17912/micropub.biology.002167

**Published:** 2026-06-24

**Authors:** Iurii Orlov, Merlin Naud, Walha Taras, Ilke Sen

**Affiliations:** 1 Mondor Institute of Biomedical Research, Créteil, IDF, FR; 2 Faculty of Medicine, Paris-Est Créteil University, Créteil, IDF, FR; 3 French Institute of Health and Medical Research, Paris, IDF, FR

## Abstract

Sarcopenia, the age-related decline in skeletal muscle mass, function, and strength, is driven by mechanisms that are incompletely understood. We analyzed a large meta-analysis of human skeletal muscle transcriptomic datasets to identify candidate regulators of sarcopenia. Orthologs of these candidate genes in *Caenorhabditis elegans* were targeted by RNA interference, and age-dependent motility was assessed by thrashing assay on days 1, 4, 7, and 11 of adulthood. Of 243 genes tested, silencing of 143 did not significantly alter motility at any time point. These results provide a reference dataset for future studies of muscle aging and sarcopenia.

**Figure 1. Selection of candidate genes for motility-based RNAi screens and representative results:** The candidate gene list was generated using results and clinical parameters from the meta-analysis published in Su et al., 2015. From 957 genes significantly associated with aging in human skeletal muscle (p < 0.05, FDR ≤ 5%), we selected genes significantly associated with physical capacity (210 genes) and type 2 diabetes (T2D; 89 genes) (p < 0.05) as potential regulators of sarcopenia and its exacerbation in T2D. Twenty-five genes were common to both lists. From 274 human genes, we identified 243 *C. elegans* orthologs for which RNAi clones were available in both the Ahringer and ORFeome v1.1 libraries. These were subjected to motility-based RNAi screening as a readout of muscle function during aging. **b)** Representative data showing two positive controls ( *eat-2* and *unc-76* ) and two representative candidate genes whose silencing did not alter worm motility across aging, days 1, 4, 7, and 11 of adulthood. Control RNAi (empty L4440 vector) data were adapted from the second screening batch and included here for illustrative purposes only. Statistical analysis for this representative dataset was performed using two-way ANOVA with Dunnett's post hoc test for multiple comparisons (*P < 0.05, **P < 0.01, ***P < 0.001, ****P < 0.0001).

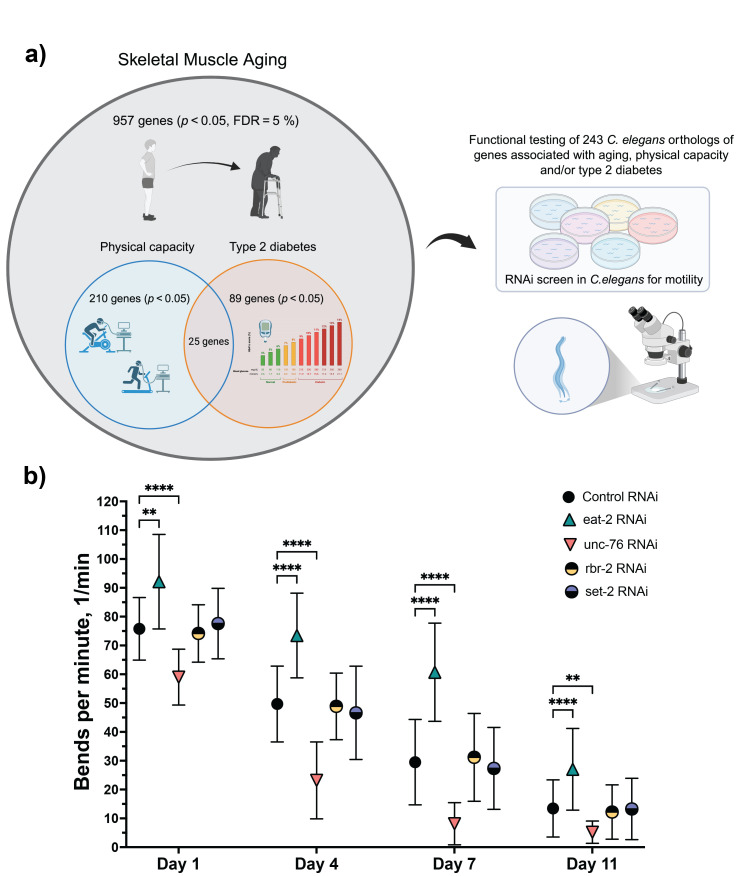



## Description

Aging is a multifaceted biological process characterized by diminished physiological coordination, reduced resilience, progressive functional decline, and increased susceptibility to age-related disorders, including cardiovascular disease, sarcopenia, and type 2 diabetes (T2D). Skeletal muscle is among the tissues most affected by aging, undergoing pronounced structural and functional changes that result in decreased muscle mass, strength, and overall performance, a condition termed sarcopenia. Sarcopenia represents a growing clinical challenge with important implications for quality of life and longevity. It arises through multiple mechanisms, including increased activity of ubiquitin ligases driving muscle protein degradation, cellular senescence, mitochondrial dysfunction, and oxidative stress during aging (Livshits & Kalinkovich, 2019; Thoma et al., 2020). Given its multifactorial nature, sarcopenia is unlikely to be effectively addressed by single-target interventions, particularly in older adults with comorbidities such as T2D (Henson et al., 2025; Leenders et al., 2013) and obesity, underscoring the need to identify new therapeutic targets (Henson et al., 2025; McColl et al., 2025).

Transcriptomic approaches provide powerful tools for the unbiased identification of genes implicated in age-related skeletal muscle decline and for elucidating the multi-pathway dysfunction that characterizes sarcopenia. Recent studies have documented widespread age-associated changes in skeletal muscle gene expression (Kedlian et al., 2024; Lai et al., 2024; Perez et al., 2022; Scott et al., 2016; Su et al., 2015; Tumasian et al., 2021). However, many of these findings remain largely descriptive and lack functional validation. Furthermore, the complex interactions among biological pathways involved in sarcopenia underscore the need for integrative approaches to prioritize candidate genes for further studies. Our recent work identified TMEM9B-AS1 as a novel regulator of skeletal muscle mass and function, downregulated in both T2D and sarcopenia (Sen et al., 2025), highlighting that additional uncharacterized factors likely contribute to muscle health under these conditions.

To systematically uncover these factors, we interrogated one of the largest publicly available meta-analyses of human skeletal muscle transcriptomic datasets, comprising approximately 3,000 samples with associated phenotypic data, including age, body mass index (BMI), physical capacity (VO₂max), and T2D status (comparison between individuals with normal glucose tolerance or T2D) (Su et al., 2015). From this dataset, we prioritized genes significantly associated with aging, physical capacity in older adults, and/or T2D (Fig. 1a), a condition in which sarcopenia is frequently observed and often exacerbated, as these factors are clinically relevant to sarcopenia and are associated with its risk and severity. (Henson et al., 2025; Leenders et al., 2013; Liu et al., 2024; Mesinovic et al., 2019). To investigate the potential roles of these genes in muscle function during aging, we performed a high-throughput RNA interference (RNAi) screen in *Caenorhabditis elegans* ( *C. elegans* ), a well-established model organism for studying conserved pathways involved in aging (Lin et al., 2018; Sen et al., 2020), sarcopenia (Gieseler et al., 2000), and metabolic disorders such as obesity and T2D (Zhu et al., 2015). *C. elegans* orthologs of human genes were identified using OrthoList 2 (Kim et al., 2018), resulting in a set of 243 candidate genes to be tested, which have RNAi clones available in Ahringer (Kamath et al., 2003)or ORFeome 1.1 (Reboul et al., 2003) libraries. Gene function was assessed using an RNAi-based motility screen in the RNAi-sensitized strain NL2099 ( rrf-3 ( pk1426 ) II). Locomotor activity was quantified using thrashing assay, in which animals were placed in liquid and the frequency of lateral body bends (bends per minute, BPM) was measured at four adult time points (days 1, 4, 7, and 11) to capture age-dependent changes in motility.

Of the 243 genes tested, RNAi-mediated silencing of 143 did not result in a statistically significant change in motility at any assessed time point (Extended Data Table 1). Mean ± standard deviation (SD), BPM values and associated statistical analyses for all genes and controls are provided in Extended Data Table 1. To minimize false-positive results, a Bonferroni correction for multiple comparisons was applied. Because the initial gene list was not manually curated, it included genes previously known to affect *C. elegans* motility (e.g., unc-76 and unc-115 ). These genes exhibited significant motility phenotypes upon RNAi silencing and therefore served as internal positive controls, supporting the sensitivity and validity of the screening approach. As additional positive controls, *eat-2* and *bec-1* were included in the RNAi screen. Representative results for the motility of the worms during aging upon RNAi silencing for two positive controls, eat-2 and unc-76 , as well as two candidate genes, rbr-2 and set-2 , that did not alter motility compared to control (empty L4440 vector) are shown in Fig. 1b.

Here, we report genes whose RNAi-mediated silencing did not significantly alter motility compared with control animals fed bacteria carrying the empty L4440 vector at any time point. The absence of detectable effects suggests that, under the conditions tested, these orthologs of predicted sarcopenia-associated candidates do not substantially influence age-dependent locomotor decline in *C. elegans .* However, given the high frequency of false negatives from *C. elegans* feeding RNAi experiments (Ahringer, 2006), we cannot exclude the possibility that some of the genes scored as negative in the screen (Extended Data Table 1) instead have a thrashing defect. Documenting these negative results provides a reference resource for future functional studies of muscle aging and may aid in prioritizing targets for investigation in mammalian systems.

## Methods

***C. elegans* strains and maintenance**

Experiments were carried out using the RNAi-sensitized strain NL2099 ( rrf-3 ( pk1426 ) II), obtained from the Caenorhabditis Genetics Center (CGC). Worms were maintained on nematode growth medium (NGM) plates at 18.5°C.

**RNAi by feeding**

Worms were fed *Escherichia coli* strain HT115 carrying dsRNA-expressing plasmids targeting genes of interest or the empty vector L4440 as a control. For screening purposes, all available RNAi clones targeting genes of interest from the ORFeome v1.1 library were used initially (Reboul et al., 2003). Genes not represented in the ORFeome v1.1 library were targeted using clones from the Ahringer library (Kamath et al., 2003). RNAi clones used in this study and their source libraries are listed in Extended Data Table 2.

RNAi bacteria were streaked onto LB agar plates supplemented with 50 µg/mL ampicillin and 10 µg/mL tetracycline and grown overnight at 37°C. Bacterial cultures were then prepared in LB medium containing 50 µg/mL ampicillin. 50 µL of 5× concentrated bacterial culture was seeded onto 12-well RNAi plates containing 1 mM IPTG and 50 µg/mL ampicillin. On day 1 of adulthood, worms were re-fed with 25 µL of 5× concentrated RNAi bacteria.

**Thrashing Assays**

Worms were synchronized by bleaching and maintained at 18.5°C. RNAi feeding was initiated at the L1 stage, with 35 worms seeded per well in 12-well plates. Two technical replicate wells were used per condition. At the late L4 stage, 5-fluoro-2′-deoxyuridine (FUDR) was added to a final concentration of 150 µM to prevent progeny production. The day following the addition of FUDR was defined as day 1 of adulthood.

Thrashing assays were performed at room temperature on days 1, 4, 7, and 11 of adulthood, spanning the adult lifespan of the animals. On each assay day, worms were washed from plates using 1 mL M9 buffer and transferred to wells containing 1 mL fresh M9 buffer. Suspensions were gently pipetted three times to distribute animals evenly. If most worms remained clustered or in contact, the plate was gently swirled to improve spacing. Animals were then allowed to acclimate for 30 s.

Thrashing behavior was recorded using a stereomicroscope equipped with a camera (Olympus SZX7 stereomicroscope with EP50 camera and 0.5× C-mount adapter U-TV0.5XC-3). For each well, two 30-s videos were captured, corresponding to the upper and lower halves of the well. Two wells were recorded per condition at each time point.

Videos were analyzed using the wrMTrck plugin (version 2011/10/31) in ImageJ (v1.54p) to extract BPM and related locomotor parameters. Output data were filtered to include only animals with a track duration >15 s and an average body area between 800 and 3000 pixels.

**Statistical analysis**

Filtered BPM values were analyzed in RStudio (2024.12.0 Build 467). Data distributions were assessed for normality using the Shapiro–Wilk test and for homogeneity of variance using Levene's test. Depending on these results, either two-way ANOVA or robust two-way ANOVA was applied.

Pairwise post hoc comparisons were performed between each gene-silencing condition and the corresponding empty L4440 vector control at each time point using contrasts of estimated marginal means derived from the fitted ANOVA model. P-values were adjusted for multiple comparisons using the Bonferroni method. Adjusted p < 0.05 was considered statistically significant.

**BioRender**

Figure 1a was created in BioRender (Sen, I. (2026) https://BioRender.com/15jie24).

## Reagents

**Table:** 

***C. elegans* strain **	**Genotype**	**Available from**
NL2099	rrf-3 ( pk1426 ) II	CGC

## Data Availability

Description: Extended Data Table 1. Internal controls and genes showing no significant RNAi effect on thrashing across all timepoints. Resource Type: Dataset. DOI: https://doi.org/10.22002/y0whz-33298 Description: Extended Data Table 2. RNAi clones, target genes, and source libraries used in this study. Resource Type: Dataset. DOI: https://doi.org/10.22002/7mvkp-c1033
